# SegResDeiT: a hybrid SegNet–ResNet-50–DeiT framework for automated cervical cancer segmentation and classification

**DOI:** 10.1186/s12880-025-02001-8

**Published:** 2025-11-17

**Authors:** Aaseegha M D, Venkataramana B

**Affiliations:** Department of Mathematics, School of Advanced Sciences, Vellore Institute of Technology, Vellore, Tamil Nadu India

**Keywords:** Cervical cancer (CC), SegNet, ResNet-50, DeiT, Deep learning, Cervical cancer cytology, Histopathology, Image segmentation, Medical AI

## Abstract

The prompt and precise identification of cervical cancer via cytology screening is essential for decreasing mortality; yet, traditional manual microscopy is impeded by subjectivity, operator tiredness, and limited throughput, resulting in diagnostic discrepancies. Conventional deep learning models have been investigated for automation; however, these methods frequently encounter difficulties in reconciling accurate spatial boundary segmentation with effective global contextual reasoning, thereby constraining their clinical precision and dependability. This paper introduces SegResDeiT, an innovative hybrid framework designed for the concurrent segmentation and classification of cervical cytology images. Our model incorporates a SegNet backbone for precise pixel-wise segmentation, a ResNet-50 encoder for hierarchical feature extraction, and a Data-efficient Image Transformer (DeiT) head for enhanced global context modelling and classification. The proposed model underwent thorough evaluation against leading benchmarks, achieving exceptional performance with an accuracy of 94.47%, precision of 95.66%, recall of 96.47%, F1-score of 96.06%, and outstanding segmentation quality, as demonstrated by a Dice coefficient of 96.06% and an IoU of 92.43%. An ablation investigation validated the collaborative impact of each architectural element, while a computational analysis illustrated a feasible equilibrium between superior performance and practical inference duration. The results unequivocally indicate that SegResDeiT outperforms current methodologies, providing a reliable and effective alternative likely to improve the precision and availability of automated cervical cancer screening, with considerable prospects for practical use.

## Introduction

Cervical cancer is a significant health concern that profoundly affects women globally. This condition arises when atypical cells in the cervix, the lowest segment of the uterus that connects to the vagina, proliferate uncontrolled [1]. As the condition advances, these atypical cells may undergo alterations such as dysplasia or cervical intraepithelial neoplasia (CIN), potentially resulting in serious health issues if not properly addressed. The ramifications of cervical cancer may be severe. If left untreated, the aberrant cells may disseminate to other regions of the body, resulting in more health problems and possibly diminishing general well-being. It is lamentable that almost 90% of these occurrences and fatalities transpired in low- and middle-income nations, where access to comprehensive healthcare services and preventative measures is often constrained [2]. This health discrepancy underscores the need to tackle cervical cancer globally and guarantee that all persons, irrespective of their socioeconomic level, have equitable access to life-saving therapies [3].

Cervical cancer ranks as the second most prevalent disease for both incidence and death among women of reproductive age worldwide, with a notably significant burden in nations with the lowest Human Development Index (HDI) [4]. The primary aetiology of cervical cancer is the persistent chronic infection by human papillomavirus (HPV), with HPV strains 16 and 18 accounting for 71% of cervical cancer cases globally [5].

The principal cause of cervical cancer is infection with the human papillomavirus (HPV), specifically the high-risk variants. Human Papillomavirus (HPV) is a widespread sexually transmitted virus, with estimates suggesting that the majority of persons will be exposed to it at some stage in their life [6]. Although the majority of HPV infections spontaneously resolve without consequences, in certain instances, the virus may remain and induce atypical cellular alterations in the cervix [7]. Over time, these alterations may progress into cancerous growths. Multiple factors influence the likelihood of acquiring cervical cancer. In addition to HPV infection, variables include smoking, absence of regular Pap or HPV screenings, immune system impairment, and extended use of oral contraceptives, particularly birth control, which might elevate the risk of acquiring this illness. Furthermore, participating in sexual relations with several partners and bearing multiple offspring are variables that must be taken into account when assessing the risk of cervical cancer [8].

Permana et al., [9] explored many investigations about cervical cancer detection using deep learning and machine learning models applied to colposcopy pictures. Challenges persist in establishing standard colposcopic pictures and mitigating the interference of reflected light in the vicinity of cervical lesions. This work will assist researchers in implementing realistic and effective pre-processing, feature extraction, segmentation, and classification techniques.

This study utilises advanced approaches in image segmentation to tackle this difficulty. Image segmentation and clasation are integral to our research, functioning as a means to accurately delineate and identify regions impacted by cervical cancer in medical imaging. The architectural architecture of the proposed framework is systematically based on three complementing deep learning models, each chosen for its unique strengths in medical image processing.

SegNet serves as the segmentation backbone owing to its efficient encoder-decoder design with pooling indices, which maintains spatial information essential for accurate boundary delineation of epithelial tissues. ResNet-50 is utilized as a feature improvement module, employing its deep residual connections to mitigate disappearing gradients and extract intricate hierarchical features that differentiate delicate morphological patterns between normal and cancerous cells. The Data-efficient Image Transformer (DeiT) is utilized to collect long-range contextual dependencies via its distilled self-attention method, facilitating the model’s comprehension of global tissue organization and cellular relationships essential for precise classification. This tri-component synergy facilitates local feature extraction and global context modelling, yielding a comprehensive approach for cervical cancer diagnosis.

### Contribution of the study

- The novelty lies in the unique integration of SegNet, ResNet-50, and DeiT into a single, end-to-end framework specifically designed for the dual task of cervical cancer segmentation and classification, which has not been previously explored.
- The framework introduces a novel multi-task learning architecture with a shared encoder and dual decoders, ensuring feature learning is mutually beneficial for both precise boundary segmentation and accurate image-level classification.
- The strategic use of the Data-efficient Image Transformer (DeiT) addresses the critical challenge of limited dataset sizes in medical imaging, making advanced transformer-based attention practical for this domain.
- The ablation study provides empirical evidence that the synergistic combination of these components yields superior performance compared to using them in isolation or against modern architectures like Swin-UNet, validating the integration strategy.
- The primary contribution is a purpose-built, effective hybrid solution that demonstrates a significant practical advancement for cervical cancer diagnosis, rather than a claim of fundamental algorithmic novelty.

### Organization of the paper

The subsequent sections of this document are organised as follows: Section II pertains to existing research on the segmentation and categorisation of cervical cancer. Section III gives the background of the suggested models. Section IV delineates the suggested methodological procedure. Section V examines the study’s results and evaluates the efficacy of the proposed strategy. Section VI concludes this research and outlines future prospects.

## Related work

Wang et al. (2024) conducted an extensive evaluation of CT, MR, and PET image segmentation on cervical cancer. They emphasized that residual modules and squeeze-and-excitation (SE) blocks augment model performance, while enhanced level set methods provide superior segmentation accuracy relative to some classical techniques [10]. Shaik et al. (2023) introduced a multi-resolution fusion deep convolutional network that concurrently executes segmentation and classification on Pap smear pictures with notable efficiency and precision (IoU 0.83 for segmentation, 90% classification accuracy). Their multi-task learning approach illustrated the advantages of concurrent optimization in cervical cancer screening [11].

Mathivanan et al. (2024) established a framework based on MobileNetv2-YOLOv3 for classifying cervix types and detecting cancer in colposcopy images, achieving a mean average precision (mAP) of 99.88% and a classification accuracy of over 94%. This underscores the application of lightweight models suitable for therapeutic environments [12] .

Sarhangi et al. (2024) examined deep learning segmentation and classification methodologies, highlighting the application of architectures such as U-Net, ResNet34, DenseNet121, and ensemble learning for segmenting cytoplasm and nucleus in cervical cytology images. Lightweight attention networks, such as LFANet, and dual-supervised sampling networks, like DSSNet, reduce processing demands while maintaining segmentation precision [13].

Himabindu et al. (2025) presented a Leveraging Swin Transformer with Ensemble Deep Learning Approach (LSTEDL-CCS) for the segmentation and classification of colposcopy images. The application of Wiener filtering for preprocessing and feature extraction via the Swin Transformer significantly enhanced segmentation quality and detection efficacy [14].

Abinaya et al. (2024) introduced an innovative cervical cancer classification system that integrates a 3D convolutional neural network with Vision Transformer (ViT) modules and a kernel extreme learning machine (KELM) classifier, attaining a remarkable accuracy of 98.6%. Their model effectively recovers spatiotemporal data pertinent to lesion segmentation and classification [15]. Mehedi et al. (2024) conducted a comparative analysis of CNN and ViT models in cervical cancer diagnosis, observing that although CNNs exhibit comparable performance, ViTs demonstrate superior capability in managing intricate image features, indicating the potential for hybrid architectures [16]. Furthermore, innovative lightweight multi-scale feature fusion segmentation models have been created to segment cervical lesions in colposcopy pictures quickly, therefore improving screening accuracy and minimizing computational demands [17].

This study introduces Iterative Pseudo-labelling Based Adaptive Copy-Paste Supervision (IPA-CP) for semi-supervised CT tumour segmentation [18]. The problem of segmenting tiny tumours was underestimated in prior investigations on larger organs. The IPA-CP method refines predictions by incorporating prediction disparities between teacher and student models using two-way uncertainty-based adaptive augmentation and an iterative pseudo-label transition [19]. Extensive studies on in-house and public datasets reveal that IPA-CP outperforms state-of-the-art semi-supervised segmentation algorithms, particularly for small tumour segmentation, suggesting potential medical applications [20].

Pacal et al. (2025) assessed deep learning models for the segmentation and classification of Pap smear images, integrating multiple CNN and Vision Transformer methodologies that attained detection accuracies exceeding 99%. Self-supervised learning, federated learning, and graph neural networks enhance segmentation and classification methodologies beyond traditional supervised CNN or ViT models, effectively tackling data scarcity and privacy issues in medical imaging [21].

## Background

### SegNet model

**Fig. 1:**
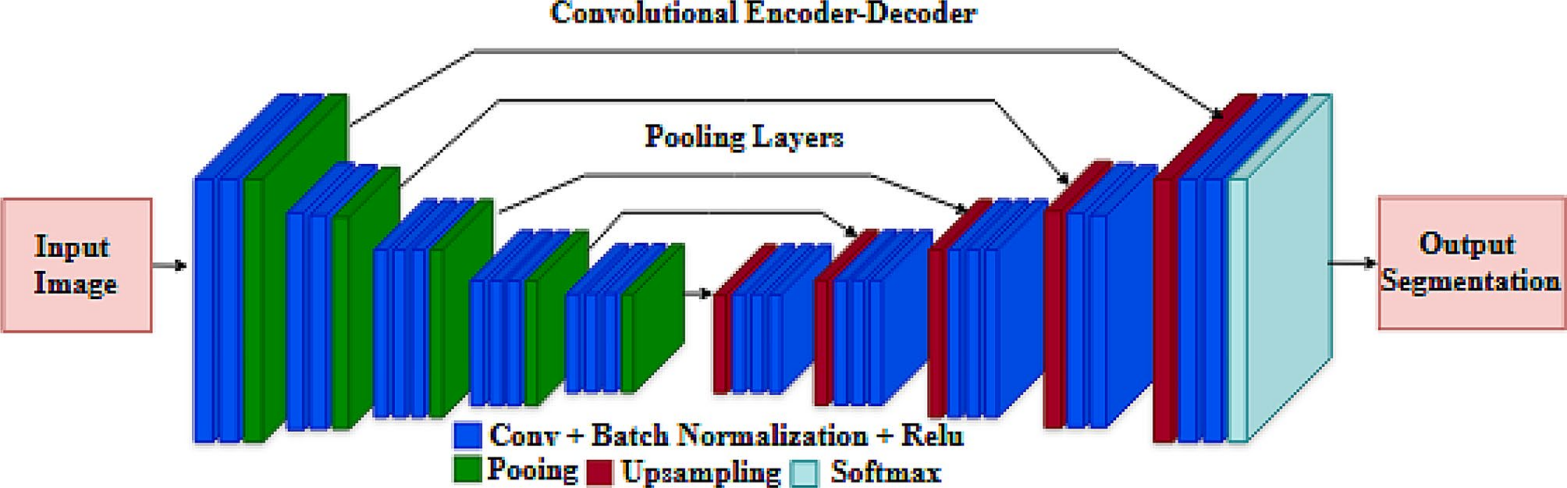
Architecture of the SegNet model

The SegNet model, an effective encoder-decoder architecture shown in Fig. 1, is designed explicitly for pixel-wise segmentation tasks [22]. The encoder extracts high-level feature representations from the input picture, while the decoder reconstructs these features to create the segmentation mask. SegNet distinguishes itself from CNN architecture by using transposed convolutional layers in the decoder [23].

The input dimensions of the SegNet model are 224 × 224 × 1, since it operates with greyscale images. The encoder consists of three blocks, each including two layers of 2D convolutional and max-pooling layers with progressively larger filter sizes. The filters in every convolutional layer measure 3 × 3. The ReLU activation function is implemented for non-linearity after each convolution. The convolutional layer extracts features, while the pooling layers, using a stride of 2 × 2, diminish the resolution of these features, enabling the method to concentrate on the most significant high-level features and thereby reduce computing time.

The decoder employs transposed convolutional layers to upsample the feature maps. The feature maps are immediately sent from the encoder to the corresponding decoder layer via the skip connection. The decoder’s last layer is a 1 × 1 convolutional layer, used for binary segmentation using a sigmoid activation function [24].

### ResNet50

The residual neural network (ResNet) was developed by He et al. [25]And won the ImageNet Large Scale Visual Recognition Challenge (ILSVRC 2015). By introducing residual connections between layers, ResNet improves performance during the training process, minimizes loss, and preserves knowledge gain. An output of a layer that has a residual link is a convolution of its input plus its input [26].

**Fig. 2:**
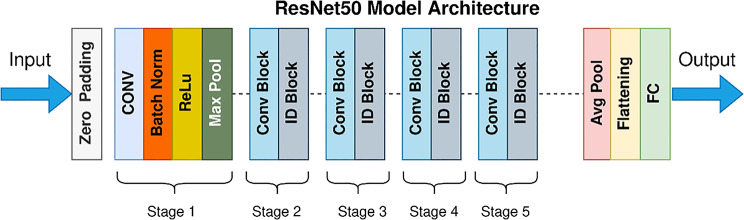
Architecture of the ResNet50 model

The ResNet 50 architecture comprises convolutional layers, pooling layers, batch normalization, average pooling, a fully connected layer, and a Softmax function. The model is trained using a diverse array of parameters, and their weights are modified throughout 50 epochs [27]. The architecture of ResNet50 is shown in Fig. 2.

### Data-efficient image transformer (DeiT)

**Fig. 3:**
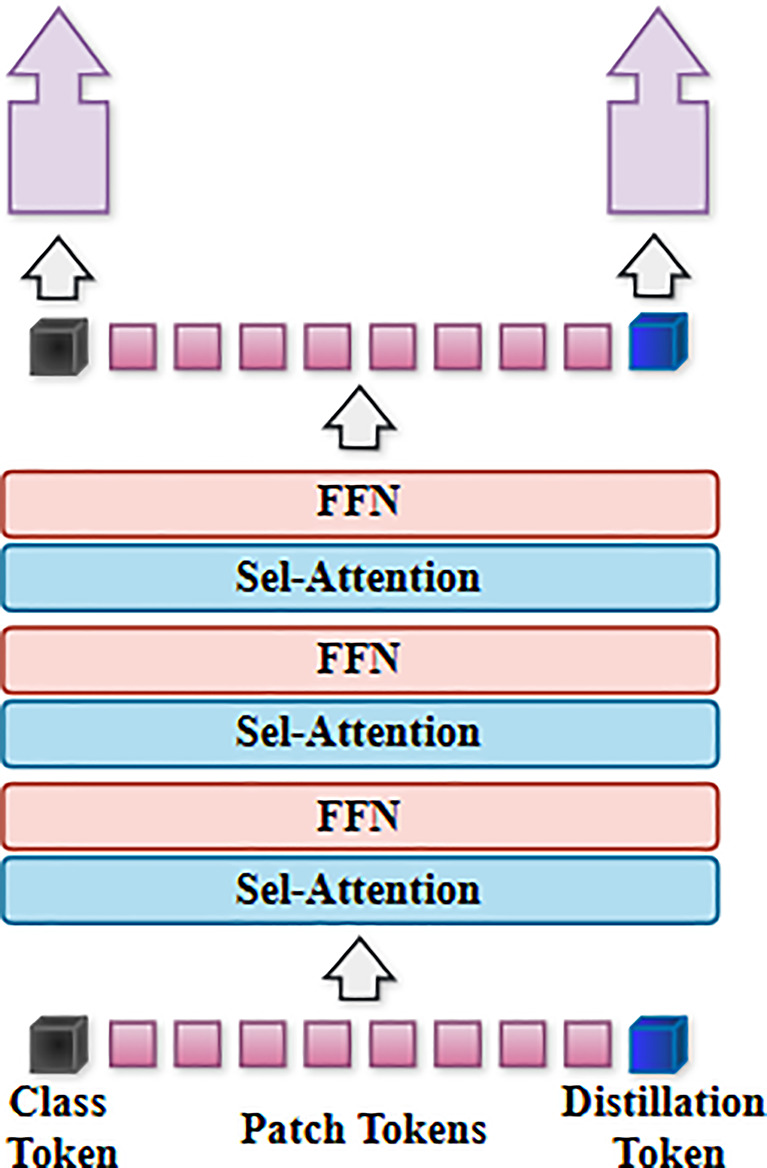
Architecture of the Data-efficient image Transformer (DeiT)

While the ViT model attains state-of-the-art performance, the DeiT article has uncovered several shortcomings of the ViT model. A substantial dataset is used for pre-training on JFT-300 M, followed by fine-tuning on ImageNet. Secondly, the JFT-300 M dataset used for transfer learning is a private dataset of Google. It is not accessible for individual usage. Thirdly, the required training duration is considerable. the DeiT model shown in Fig. 3 has been designed to address these challenges [28]. The DeiT model preserves ViT’s architecture while modifying the training methodology to achieve enhanced performance on the ImageNet dataset, without relying on extensive datasets.

A distillation procedure tailored for the transformer architecture using distillation tokens. Incorporating Distillation Tokens into the initial embedding values supplants the function of the Special Classification (CLS) token. This token interacts with other embeddings through self-attention and produces a modified value. The Class Token and Distillation Token have been verified as distinct vectors [29]. As training progresses, the cosine similarity increases, and information from the Teacher Model is conveyed via distillation to produce the final result. Research has shown that the Teacher Model has a substantial influence on the efficacy of distillation [30]. Due to these distillation techniques, DeiT exhibits much superior performance compared to the ViT model on a very modest biomedical dataset. This enhances the likelihood of using a relatively little dataset [31]. 

## Proposed methodology

### Dataset and description

All data generated or analysed during this study are included in this published article “Cell Segmentation in Digitized Pap Smear Images Using an Ensemble of Fully Convolutional Networks“ [32]. In this study the data was collected from the publicly available dataset from the following website: https://osf.io/cka2f/wiki/home/. The APACS23 dataset contains a total of 3,565 digitized Pap smear images originating from 5 specimens. The images were captured using a 3DHistec Panoramic 1000 digital slide scanner, equipped with an Adimec Q-12 A-180Fc bright-field camera. During the scanning process, 20x magnification was used. All the images have the exact dimensions of 2000 × 2000 pixels and are split into training and test sets. The original APACS23 training set containing 2,227 images was further split into training (80%, 1,782 images) and validation (20%, 445 images) sets using stratified sampling to maintain class distribution. The pre-defined test set of 1,338 images remained untouched for final evaluation. This splitting strategy ensured proper hyperparameter tuning and model selection while maintaining unbiased performance assessment on the hold-out test set. This standardised structure enables the consistent construction and evaluation of models, facilitating strong benchmarking of the proposed SegResDeiT framework.

### Data preprocessing

#### Image normalization

All raw photos in the APACS23 collection are in JPEG format, with high-resolution dimensions of 2000 × 2000 pixels, and were obtained using bright-field microscopy at 20x magnification. Before model training, each image was normalized to guarantee uniform intensity distribution throughout the dataset. Pixel values were normalized to a predetermined range of 0 to 1 using min-max normalization. This normalization alleviates discrepancies in lighting conditions and staining variations among samples, which is crucial for deep learning models to generalize effectively across patient slides. Normalization was independently applied to each picture channel to maintain the structural integrity of the tissue.

#### Mask preparation and label encoding

Every image in the collection is linked to a manually annotated binary segmentation mask that defines the aberrant epithelial areas. The masks were loaded and preprocessed to conform in size and alignment with the photos. A binary label encoding method was utilized, assigning pixel values of 1 to aberrant regions (foreground) and 0 to the background. This binary representation allows the model’s segmentation module to do pixel-wise classification between normal and potentially cancerous tissue. In classification tasks, image-level labels were assigned according to the presence or absence of positive pixels in the segmentation masks: images with any non-zero regions were classified as containing aberrant tissue, whilst those with zero-valued masks were classified as normal.

#### Image resizing and patch extraction

Resizing was necessary to decrease computing expenses while maintaining significant morphological characteristics, owing to the original photos’ high resolution. All photos and their respective masks were scaled to a uniform dimension of 256 × 256 pixels, employing bilinear interpolation for the images and nearest-neighbour interpolation for the masks to prevent label distortion. Alongside resizing, patch-based extraction was employed to direct the model’s focus on specific areas of diagnostic significance, particularly in instances when aberrant regions were infrequent. Patches were excised with overlap to ensure that tiny lesions near the image margins were included, hence preserving sensitivity and contextual integrity.

#### Data augmentation

A comprehensive data augmentation method was implemented to mitigate potential class imbalance and enhance the model’s resilience. Geometric and photometric changes were applied to both the training images and their corresponding masks. The alterations encompassed random rotations, horizontal and vertical flips, zooming, and brightness modifications. Data augmentation was conducted in real-time during training to create a varied learning environment, thus enhancing the model’s generalizability to novel data. Augmented samples preserved their original semantic content while injecting variability that mimics diverse imaging settings and tissue orientations.

#### Dataset partitioning and preprocessing pipeline

The APACS23 dataset is partitioned into training and test sets containing 2,227 and 1,338 pictures, respectively. No additional stratified sampling was conducted, as the established division guarantees a uniform norm for comparison assessment. All preparation procedures normalization, resizing, mask alignment, and augmentation were executed through a cohesive pipeline developed with Python modules including OpenCV, NumPy, and Albumentations. This pipeline guaranteed the synchronization of each image-mask pair throughout all changes. The final preprocessed dataset was saved in NumPy array format to enable rapid batch loading during model training and evaluation.

### Model building

#### SegResDeiT architecture overview

The SegResDeiT framework is a hybrid deep learning model that integrates the advantages of SegNet, ResNet-50, and the Data-efficient Image Transformer (DeiT) for the automatic segmentation and classification of cervical cytology pictures. This dual-branch design processes input images via a shared encoder and splits into two complimentary tasks: pixel-wise segmentation and image-level classification. The segmentation branch utilizes SegNet’s spatial accuracy, whilst the classification branch leverages ResNet-50’s hierarchical abstraction and DeiT’s contextual analysis. The amalgamation of these components guarantees that both tasks derive advantages from common spatial and semantic representations, resulting in improved diagnostic efficacy.

#### Segmentation module with SegNet backbone

The segmentation pathway is developed via an adapted SegNet architecture. The encoder consists of many convolutional blocks interspersed with batch normalization and ReLU activations, succeeded by max pooling layers that downsample feature maps while preserving spatial indices. These pooling indices are essential during decoding, as they direct the upsampling process to rebuild object boundaries accurately. The decoder replicates the encoder’s architecture and reinstates spatial resolution utilizing the preserved indices, succeeded by convolutional refinement. This framework enables the model to retain the intricate characteristics of atypical epithelial areas and minimise the loss of boundary information typically observed in dense tissue clusters. The output segmentation mask is generated using a sigmoid activation function to create a pixel-wise probability map. The segmentation branch is governed by a composite loss function that integrates Binary Cross-Entropy (BCE) and Dice Loss, expressed as:

1 $${{\cal L}_{seg}}\> = \>{\lambda _1}\> \times \>{{\cal L}_{BCE}}\, + \>{\lambda _2}\> \times \>{{\cal L}_{DICE}}$$

Where $\:{\mathcal{L}}_{BCE}$ represents Binary Cross-Entropy loss:

2 $${{\cal L}_{BCE}} = - {1 \over N}\sum\nolimits_{i = 1}^N {\left[ {{g_i}{\rm{log}}\left( {{p_i}} \right) + (1 - {g_i}){\rm{log}}(1 - {p_i})} \right]} $$

where $\:\lambda_{1}\:and\:\lambda_{2}$ are the weighting coefficients. The Dice Loss is articulated as follows:

3 $${{\cal L}_{DICE}} = 1 - {{2\sum\nolimits_{i = 1}^N {{p_i}{g_i}} + \epsilon } \over {\sum\nolimits_{i = 1}^N {{p_i}} + \sum\nolimits_{i = 1}^N {{g_i}} + \epsilon }}$$

In this context, $\:{p}_{i}$ and $\:{g}_{i}$ denote the expected and actual values at pixel $\:i,$ respectively, while $\:ϵ$ serves as a smoothing constant to prevent division by zero.

#### Feature extraction using ResNet-50

ResNet-50 is utilized as a standard feature extractor to augment the model’s representational capacity for both segmentation and classification tasks. The deep residual network obtains intermediate feature maps from SegNet’s encoder and processes them via a series of identity and convolutional shortcut blocks. This design allows the network to acquire deep representations without deterioration, enhancing the detection of nuanced differences in cellular structure. The multi-tiered characteristics derived from ResNet-50 are transmitted to both the decoder and the transformer classifier. The residual features enhance semantic depth for segmentation refinement and offer a concise and expressive representation for further global reasoning.

#### Transformer-based classification with DeiT

The classification branch utilizes the Data-efficient Image Transformer (DeiT) to gather global dependencies and contextual clues vital for comprehensive image classification. The output features from ResNet-50 are restructured into non-overlapping patches and linearly inserted into a sequence appropriate for transformer input. DeiT utilizes multiple self-attention layers to analyze these patch embeddings, enabling the model to infer relationships across remote areas of the image. A trained classification token summarizes global information, which is processed through a multi-layer perceptron head for binary classification. The classification branch is trained with the Binary Cross-Entropy Loss, defined as:

4 $${\mathcal{L}}_{CLS}=-\left[y\cdot\text{log}\widehat{\left(y\right)}+\left(1-y\right)\cdot\text{log}\left(1-\widehat{y}\right)\right]$$

where 𝑦 represents the ground truth label and 𝑦̂ is the projected probability. This framework penalizes erroneous predictions and directs the model towards accurate differentiation between normal and pathological samples.

#### Loss weight ablation

The loss weighting parameters (λ₁, λ₂, α) were established by a methodical grid search on the validation set. We assessed combinations where λ₁ + λ₂ = 1.0 for the segmentation loss equilibrium and α ∈ [0.5, 1.0, 1.5, 2.0] for the classification weight. The ideal configuration was determined to be λ₁ = 0.6, λ₂ = 0.4, and α = 1.0. This weighting prioritizes Binary Cross-Entropy somewhat above Dice loss to ensure effective probability calibration while utilizing Dice’s advantages in addressing class imbalance. The identical weighting (α = 1.0) between segmentation and classification tasks facilitated balanced optimization, preventing either job from overshadowing the gradient updates.

#### Multi-task learning and loss optimization

The model employs a multi-task learning technique that concurrently optimizes segmentation and classification objectives. The comprehensive loss function is delineated as:

5 $${\mathcal{L}}_{total}={\mathcal{L}}_{seg}+\:\alpha\cdot{\mathcal{L}}_{cls}$$

where $\:\alpha\:$ is a balance parameter that regulates the impact of classification loss on the overall target. Joint optimization ensures that shared layers acquire representations that are concurrently beneficial for both local border prediction and global category inference, thereby improving the model’s coherence and generalization.

#### Training configuration and implementation specifications

The SegResDeiT model was implemented using the PyTorch deep learning framework. All input images and their corresponding masks were resized to 256 × 256 pixels prior to training. To enhance data variability and mitigate overfitting, a comprehensive set of augmentation techniques, including random rotations (± 15°), horizontal and vertical flips, zoom (0.8–1.2×), and brightness adjustments (0.8–1.2×) were applied dynamically during training using the Albumentations library. The model was trained on a workstation equipped with an NVIDIA RTX 3080 GPU (10GB VRAM). Due to hardware memory constraints, a batch size of 8 was utilized; however, gradient accumulation over 2 steps was employed to simulate an effective batch size of 16, thereby stabilizing gradient estimates. The Adam optimizer was used with an initial learning rate of 1 × 10⁻⁴ and a weight decay of 1 × 10⁻⁵. A cosine annealing scheduler was implemented to gradually reduce the learning rate, facilitating smoother convergence. The training regimen spanned 30 epochs, with early stopping based on validation loss (patience = 10) to prevent overfitting. Batch normalization layers were consistently used throughout the network to ensure training stability, while dropout layers (rate = 0.3) were incorporated in the decoder and transformer classifier heads to enhance generalization. The model was trained in an end-to-end fashion using the optimized loss weights (λ₁=0.6, λ₂=0.4, α = 1.0) determined through grid search, enabling concurrent optimization of both segmentation and classification branches and promoting synergistic learning across tasks.

**Table 1:** Algorithm for SegResDeiT framework for cervical cytology analysis

Algorithm: SegResDeiT Framework for Cervical Cytology Analysis	
1.	**Input**: APACS23 dataset with images I and masks M
2.	**Output**: Segmentation masks S and classification labels C
3.	**Procedure** DATA PREPROCESSING(*I*,* M*)
4.	Normalize pixel values: I_norm_ = (I – I_min_)/(I_max−_I_min_)
5.	Resize images and masks to 256 × 256 using bilinear and nearest-neighbor interpolation respectively.
6.	Extract overlapping patches from regions of interest.
7.	Apply augmentation A = {rotation, flip, zoom, brightness adjustment}
8.	Encode masks: M_binary_ = 1 for abnormal, 0 for normal
9.	**end procedure**
10.	**Procedure** MODEL ARCHITECTURE(I_preprocessed_)
11.	**Shared Encoder**:
12.	SegNet encoder blocks with preservation of max pooling indices.
13.	ResNet-50 feature extraction with skip connections
14.	**Segmentation Branch**:
15.	SegNet decoder with unpooling using stored indices.
16.	Output: S = σ(_Conv1 × 1_(features))
17.	**Classification Branch**
18.	DeiT transformer processing ResNet-50 features.
19.	Patch embedding and self-attention computation
20.	Output: C = MLP(cls_token)
21.	**end procedure**
22.	**Procedure** TRAINING
23.	Initialize weights using He initialization
24.	Define loss functions:
25.	$\:{\mathcal{L}}_{seg}\:=\:\lambda_{1}\:\times\:\:{\mathcal{L}}_{BCE}+\:\lambda_{2}\:\times\:{\mathcal{L}}_{DICE}$
26.	$\:{\mathcal{L}}_{DICE}=1-\frac{2{\sum\:}_{i=1}^{N}{p}_{i}{g}_{i}+ϵ}{{\sum\:}_{i=1}^{N}{p}_{i}+{\sum\:}_{i=1}^{N}{g}_{i}+ϵ}$
27.	$\:{\mathcal{L}}_{CLS}=-\left[y\cdot\:\text{log}\widehat{\left(y\right)}+\left(1-y\right)\bullet\:\text{log}\left(1-\widehat{y}\right)\right]$
28.	$\:{\mathcal{L}}_{total}=\:{\mathcal{L}}_{seg}+\:\alpha\:\cdot\:{\mathcal{L}}_{cls}$
29.	Optimize using Adam (η = 10^− 4^, weight decay = 10^− 5^)
30.	Apply cosine annealing learning rate scheduling.
31.	Early stopping based on validation loss
32.	**End procedure**
33.	**Procedure** EVALUATION ( S, C, M_test_, Y_test_)
34.	Compute segmentation metrics:
35.	$\:DSC=\frac{2\left|P\cap\:G\right|}{\left|P\right|+\left|G\right|}$
36.	$\:{I}_{o}U=\frac{\left|P\cap\:G\right|}{\left|P\cup\:G\right|}$
37.	Compute classification metrices:
38.	$\:Accuracy\text{\hspace{0.17em}}=\text{\hspace{0.17em}}\frac{TP\text{\hspace{0.17em}}+\text{\hspace{0.17em}}TN}{TN+TP+FP+FN}$
39.	$$\:{F}_{1}=\frac{2*\text{P}\text{r}ecision*\text{R}\text{e}call}{\text{P}\text{r}ecision+\text{R}\text{e}call}$$
40.	**End procedure**

### Model evaluation

The process of “model evaluation”, as shown in Table 1, examines whether a created model can be applied to new data to establish its generalizability. Accuracy, precision, recall, F1 score, are some of the numerous measures used to assess models’ ability.

### Performance metrics

**Fig. 4:**
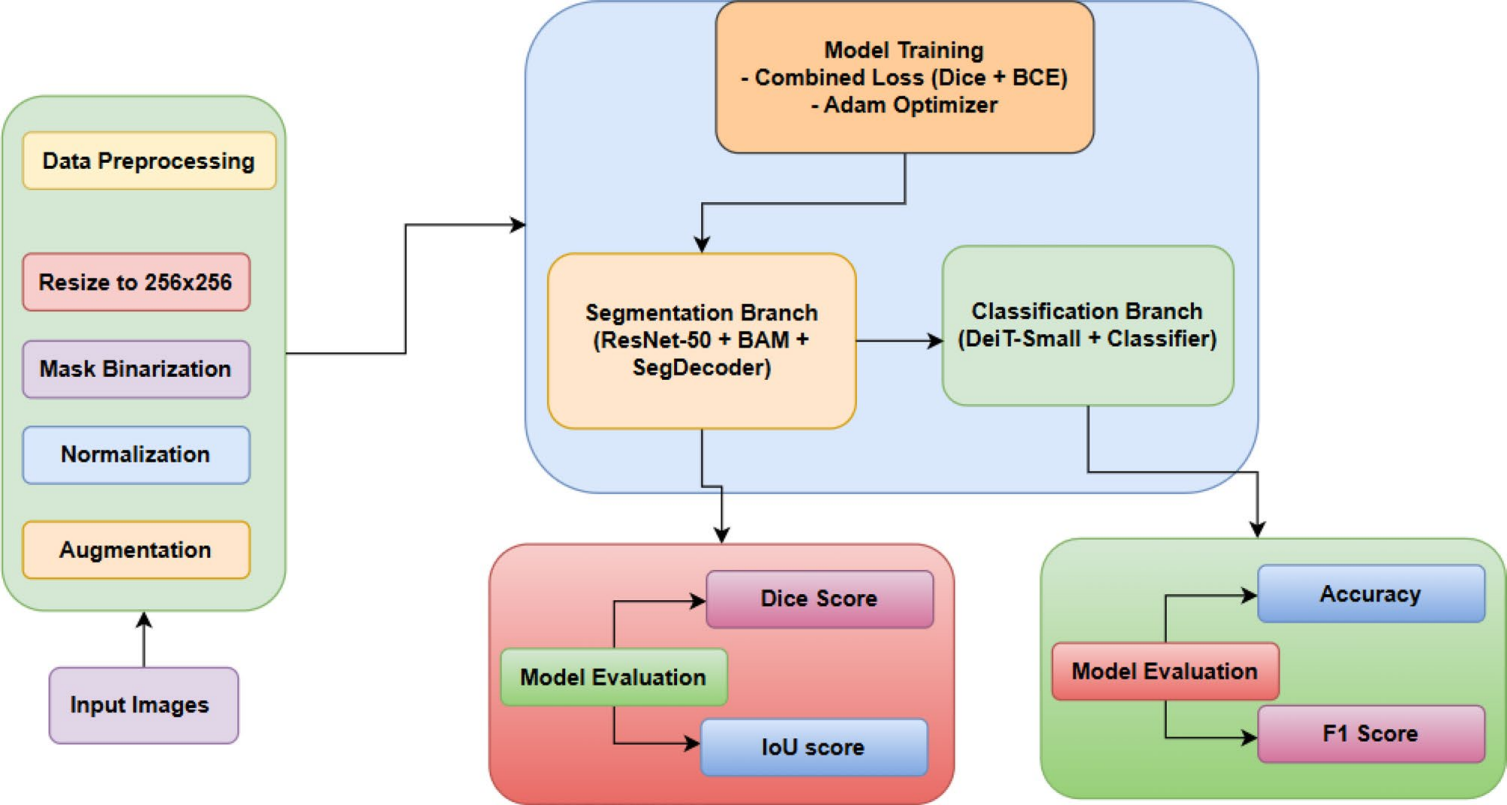
Proposed architecture

SegResDeiT was evaluated for both semantic segmentation of abnormal epithelial regions and binary classification of cytology images, using clinically relevant metrics. For segmentation, the Dice Similarity Coefficient (DSC) and Intersection over Union (IoU) were employed to assess spatial overlap and localization accuracy, which are critical for handling class imbalance and boundary delineation in medical images. High DSC and IoU scores indicate precise detection of lesions, supporting diagnostic and treatment planning. For classification, accuracy, precision, recall, and F1-score were used to measure diagnostic performance, with emphasis on recall and precision due to the clinical importance of minimizing false negatives and false positives. Figure 4 presents the proposed framework. Metrics were calculated using a different test set from APACS23 to guarantee an unbiased assessment. Ground truth masks and derived image-level labels facilitated assessment. The trained SegResDeiT model outputs both segmentation and classification in a single forward pass through multi-task learning, enhancing performance and efficiency. Its consistent results across tasks affirm its potential for integration into automated cervical cancer screening pipelines.

### Dice Similarity Coefficient (DSC)

The Dice Similarity Coefficient (DSC) is a commonly utilised metric in medical image segmentation, as it effectively quantifies spatial overlap between predicted and ground truth masks. This is especially advantageous when the tumour occupies a minor segment of the image, rendering conventional accuracy-based metrics less dependable.

The Dice coefficient is mathematically defined as:

6 $$DSC\:=\frac{\:2\:\cdot\:\:|P\:\cap\:\:G|\:\:}{\left(\right|P|\:+\:|G\left|\right)}\:=\frac{\:2TP}{(2TP\:+\:FP\:+\:FN)}$$

Where: P denotes the anticipated mask.

- G represents the ground truth mask.
- TP, FP, and FN denote the quantities of true positive, false positive, and false negative pixels, respectively.

The Dice score varies from 0 (indicating no overlap) to 1 (indicating perfect overlap). This study utilises it as the principal evaluation metric due to its equilibrium between precision and recall, as well as its heightened sensitivity to misclassification of tumour boundaries.

### Intersection over Union (IoU)

The Intersection over Union (IoU), also referred to as the Jaccard Index, quantifies the ratio of the intersection to the union of the predicted and ground truth masks. It imposes penalties for both over-segmentation and under-segmentation.

7 $$\:IoU\:=\frac{\:|P\:\cap\:\:G|}{|P\:\cup\:\:G|}\:\:\:=\frac{\:TP\:\:}{(TP\:+\:FP\:+\:FN)}$$

The Intersection over Union (IoU) offers a more rigorous assessment than the Dice coefficient, particularly in edge cases where minor false positives or false negatives can considerably influence the intersection. Although Dice is more lenient in situations of class imbalance, IoU provides a more definitive assessment of overall segmentation coherence.

#### Accuracy

The simplest way to measure how often the classifier makes correct predictions is by using accuracy. This might instead be interpreted as the proportion of all correctly predicted positive outcomes divide by the total amount of forecasts made.

8 $$\:Accuracy\text{\hspace{0.17em}}=\text{\hspace{0.17em}}\frac{TP\text{\hspace{0.17em}}+\text{\hspace{0.17em}}TN}{TN+TP+FP+FN}$$

#### Precision

In contrast to this ratio in addition to one minus from it, i.e., (1 – precision), which presents the percentage false negatives; 1/Precision yields recall.

9 $$\:Precision=\frac{TP}{TP+FP}$$

#### Recall

On other hand there are called false negatives in relation with True Negatives.

10 $$\:Recall=\frac{TP}{TP+FN}$$

#### F1-Score

The harmonic mean of the recall and accuracy scores is used to calculate it.

11 $$\:{F}_{1}=\frac{2*\text{P}\text{r}ecision*\text{R}\text{e}call}{\text{P}\text{r}ecision+\text{R}\text{e}call}$$

## Results and discussion

The following section presents and analyzes the results obtained from the proposed SegResDeiT model for automated cervical cancer segmentation and classification. Quantitative metrics such as IoU, Dice coefficient, accuracy, precision, recall, and AUC are used to evaluate the model’s performance. Comparisons with baseline architectures are provided to highlight the superiority of the proposed hybrid design. Additionally, confusion matrices and ROC curves are analyzed to assess classification reliability, while segmentation quality is demonstrated through visual outputs.

### Results

**Fig. 5:**
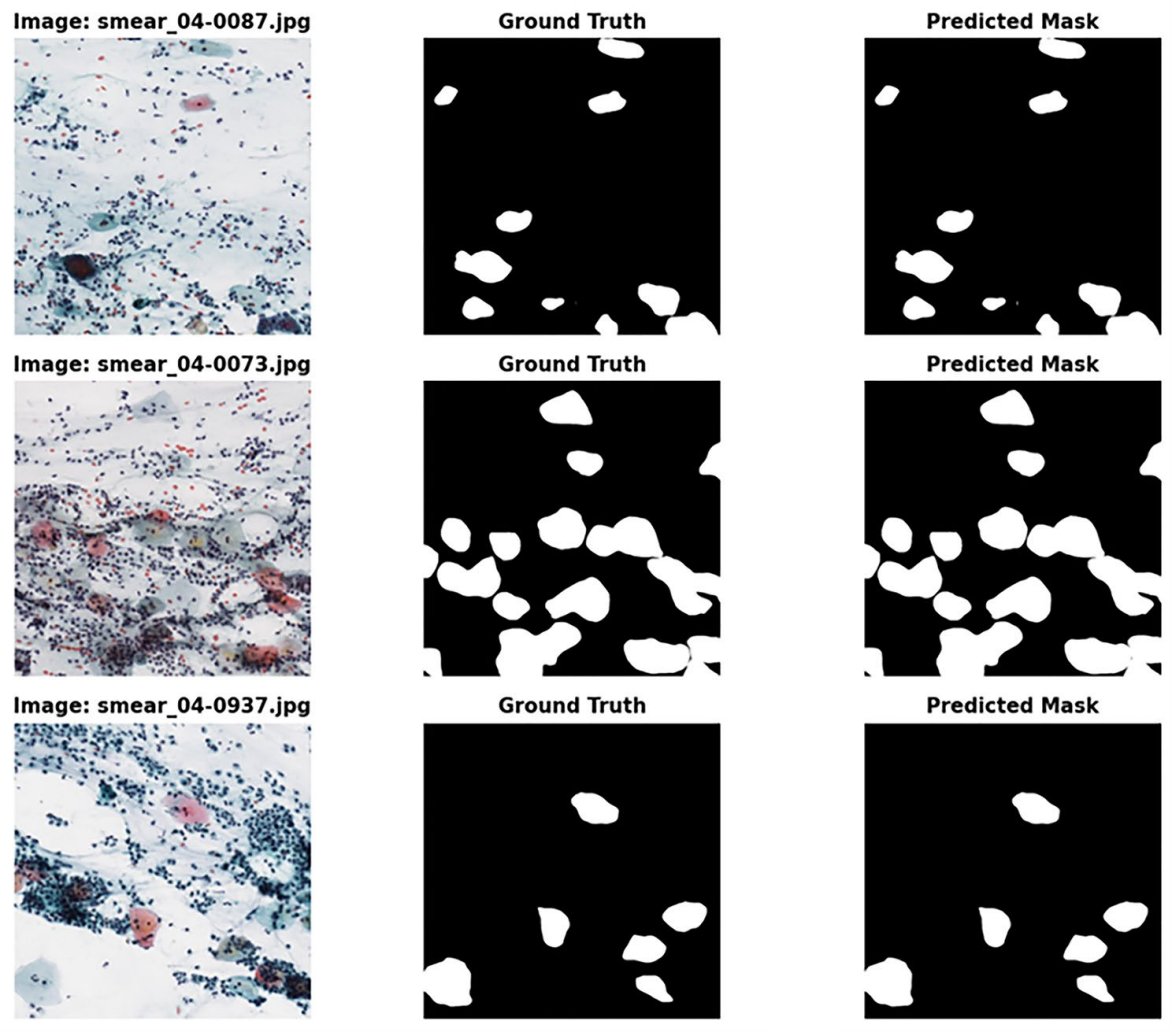
Visual comparison of ground truth and predicted tumor masks using the proposed model

The visualisation in Fig. 5 demonstrates the effectiveness of the SegResDeiT framework by displaying the input cervical cytology images alongside their ground truth masks and predicted segmentation masks. Each row comprises three elements: the original input image, the expert-annotated ground truth mask that delineates the area of cervical abnormality, and the forecast mask generated by the proposed model. The anticipated masks exhibit strong visual concordance with the ground truth, precisely delineating the lesion boundaries and regional structures while minimizing false positives and omissions. The strong alignment between the annotated and predicted regions demonstrates the model’s proficiency in acquiring detailed spatial information and its capacity to generalize effectively to novel test data. The visual uniformity across several samples underscores the reliability and accuracy of the SegResDeiT architecture in delineating clinically relevant regions, therefore affirming its utility in practical cervical cancer screening and diagnostic processes.

**Table 2:** Performance comparison of various deep learning architectures

Model	Accuracy	Precision	Recall	F1-Score	IoU	Dice Score
SegResDeiT	0.9447	0.9566	0.9647	0.9606	0.9243	0.9606
Swin-UNet	0.858	0.9099	0.8846	0.8971	0.8134	0.8971
nnU-Net	0.7922	0.873	0.8226	0.8471	0.7347	0.8471
DeepLabV3+ With (ResNet-50)	0.7728	0.8607	0.8056	0.8322	0.7127	0.8322

**Fig. 6:**
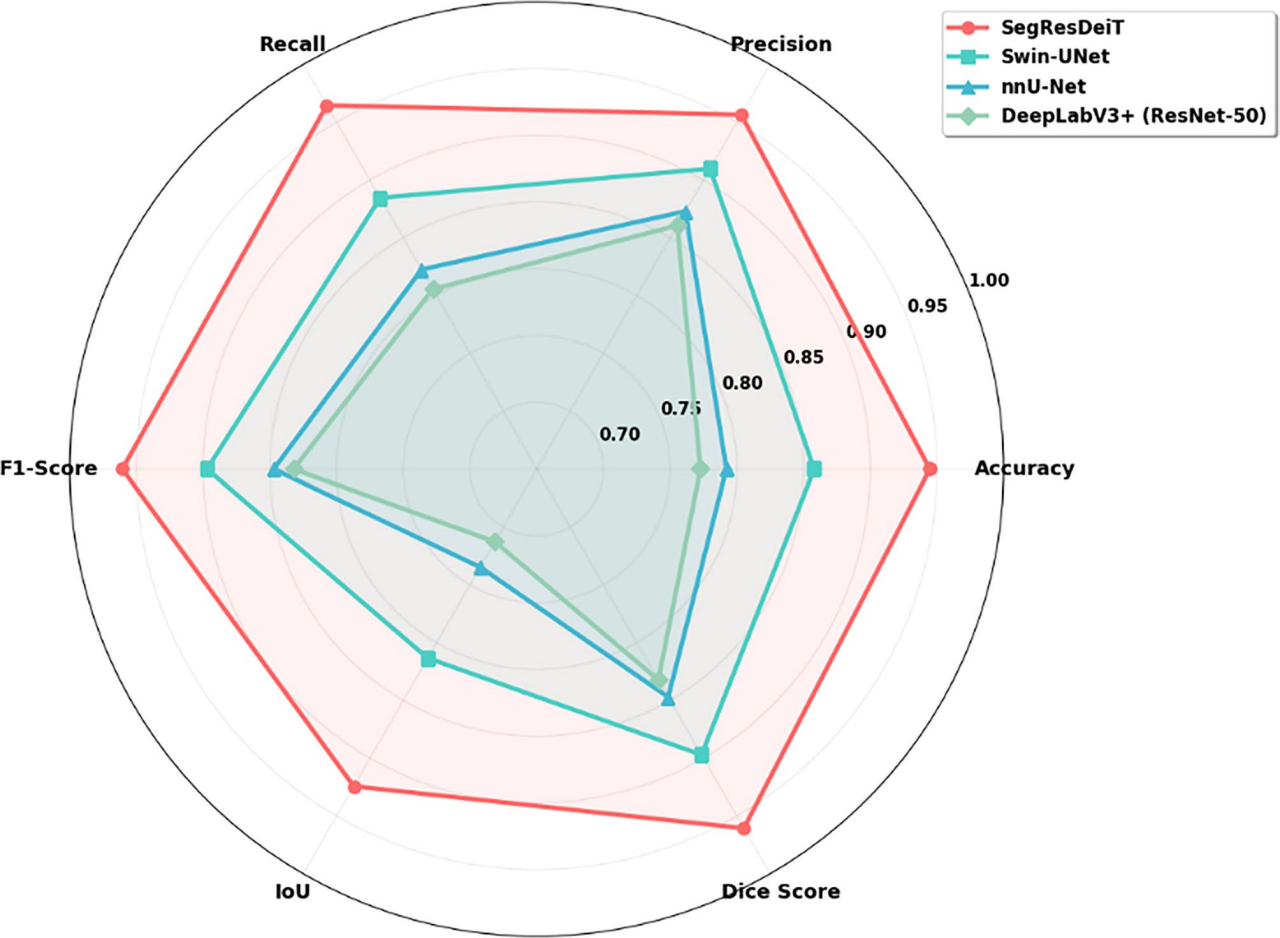
Comparative performance metrics of the evaluated models

Figure 6 shows the visual representation of the Comparative performance metrics of the evaluated models. The SegResDeiT architecture significantly outperforms the other deep learning models in every analyzed criterion, according to the performance metrics in Table 2. With an accuracy of 94.47%, precision of 95.66%, recall of 96.47%, and F1-Score of 96.06%, it obtained the most significant scores. Furthermore, with a Dice Score of 96.06% and an Intersection over Union (IoU) of 92.43%, it fared noticeably better than the alternatives in segmentation-specific metrics. With an accuracy of 85.8% and a Dice Score of 89.71%, the Swin-UNet model came in second place. In contrast, the nnU-Net (accuracy 79.22%, Dice 84.71%) and DeepLabV3+ (accuracy 77.28%, Dice 83.22%) architectures performed relatively poorly on this particular test.

### Confusion matrix

**Fig. 7:**
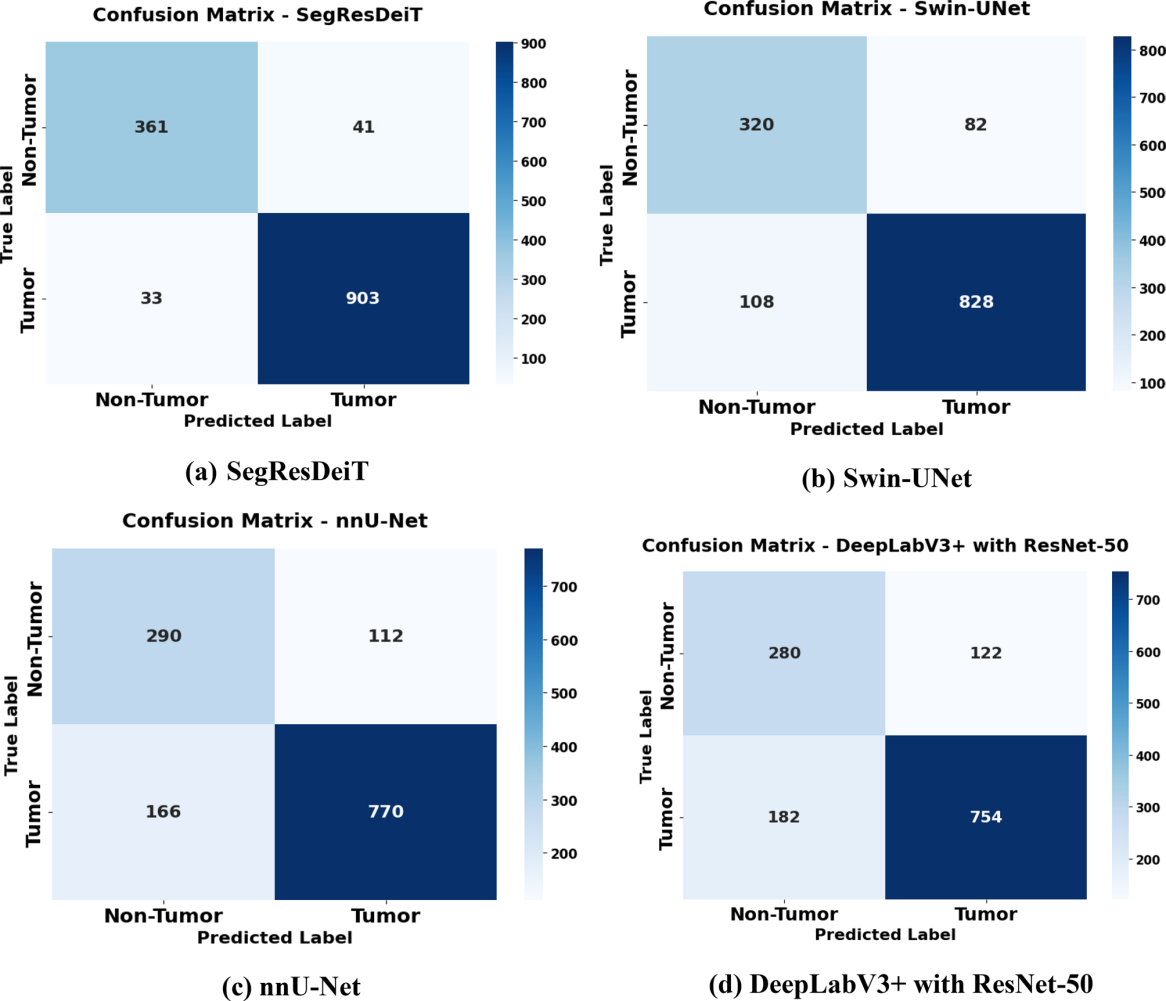
Confusion matrices of individual models compared with the proposed hybrid model, illustrating misclassification patterns. **(a)** Hybrid SegResDeiT, **(b)** Swin-UNet, **(c)** nnU-Net, and **(d)** DeepLabV3 + with ResNet-50

Figure 7 shows confusion matrices that compare the classification performance of four different segmentation architectures combined with image-level classification for tumor identification. SegResDeiT in Fig. 7 (a) misclassified 41 non-tumor samples as tumors and 33 tumor samples as non-tumors, while correctly classifying 361 non-tumor samples and 903 tumor samples. This has a balanced categorization ability and good accuracy. The model accurately detected 828 tumor samples and 320 non-tumor samples for Swin-UNet in Fig. 7 (b); however, it incorrectly classified 108 tumor instances as non-tumor and 82 non-tumor instances as tumors. The performance is marginally worse with more false positives and false negatives than SegResDeiT. The model accurately predicted 770 tumor cases and 290 non-tumor cases for nnU-Net in Fig. 7 (c). Nevertheless, it misclassified 166 tumor samples as non-tumor and 112 non-tumor samples as cancers. this indicates higher misclassification counts and a clear decline in classification performance relative to SegResDeiT and Swin-UNet. Lastly, 280 non-neoplastic and 754 tumor samples were successfully categorized using the DeepLabV3 + with ResNet-50 model in Fig. 7(d). In addition, it incorrectly identified 182 tumor samples as non-tumor and 122 non-tumor samples as cancers. With the most significant number of misclassifications in both categories, this model performs the worst out of the four. According to the confusion matrices, SegResDeiT performed the best overall, followed by Swin-UNet. In contrast, nnU-Net and DeepLabV3 + with ResNet-50 performed worse due to more erroneous predictions.

### ROC curve

**Fig. 8:**
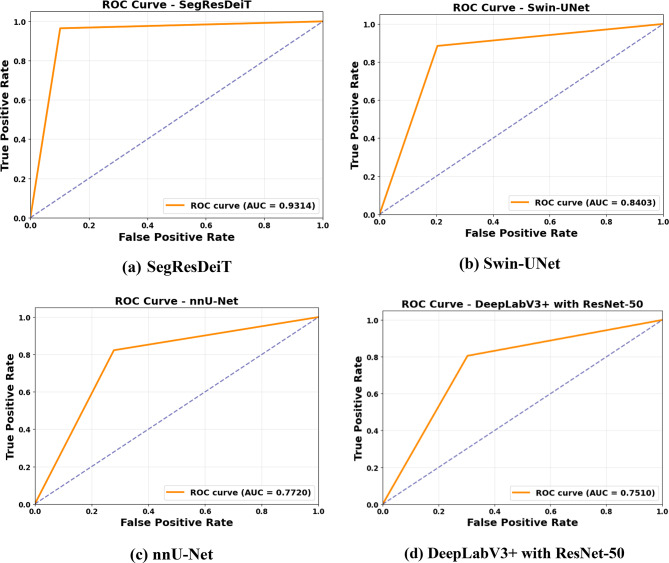
Receiver operating characteristic (ROC) curves comparing the classification performance of four models: (**a**) Hybrid SegResDeiT, (**b**) Swin-UNet, (**c**) nnU-Net, and (**d**) DeepLabV3 + with ResNet-50. The hybrid model achieved the highest AUC values across all tissue classes, demonstrating superior discriminative ability compared to the individual architectures

Figure 8 presents ROC curves that demonstrate the classification efficacy of four distinct segmentation-classification hybrid models in differentiating between tumor and non-tumor instances. 

The images show the ROC curves for SegResDeiT, Swin-UNet, nnU-Net, and DeepLabV3 + with ResNet-50 deep learning segmentation models. Each graphic compares the True Positive Rate (TPR) and False Positive Rate (FPR) for various threshold values, using the Area Under the Curve (AUC) as the main performance metric. Higher AUC values suggest better class discrimination by the model. The ROC curve in Fig. 8(a) (SegResDeiT) exhibits outstanding classification performance with an AUC of 0.9314. The curve, which hovers around the top-left corner, indicates excellent sensitivity and specificity. SegResDeiT appears to identify most genuine positives and minimize false positives. Based on its metrics (Accuracy = 0.9447, Precision = 0.9566, Recall = 0.9647, F1-Score = 0.9606, IoU = 0.9243, Dice = 0.9606), SegResDeiT is the best model across various evaluation criteria. Figure 8(b) (Swin-UNet) shows a reasonably robust ROC curve with 0.8403 AUC. The curve is flatter than SegResDeiT, indicating some compromise in differentiating positive and negative classes. The model still has competitive values: Accuracy = 0.8580, Precision = 0.9099, Recall = 0.8846, F1-Score = 0.8971, IoU = 0.8134, Dice = 0.8971. Swin-UNet, despite not surpassing SegResDeiT, is a solid model for segmentation tasks, especially when speed and accuracy are acceptable trade-offs. Figure 8(c) (nnU-Net) shows the ROC curve with an AUC of 0.7720, showing lower classification performance than the previous two models. Flatter curves indicate that the model fails to balance sensitivity and specificity. This is supported by performance metrics: Precision = 0.8730, Recall = 0.8226, F1-Score = 0.8471, IoU = 0.7347, Dice = 0.8471. Despite its good segmentation findings, nnU-Net lacks resilience and generalization. Figure 8(d) shows DeepLabV3 + with ResNet-50 has the worst ROC curve, with an AUC of 0.7510. The curve’s linearity shows low discrimination. Other metrics like Accuracy = 0.7728, Precision = 0.8607, Recall = 0.8056, F1-Score = 0.8322, IoU = 0.7127, and Dice = 0.8322 as it confirms its lower performance relative to competitors. This suggests that DeepLabV3 + can segment moderately but not precisely. The ROC study shows that SegResDeiT outperforms Swin-UNet, nnU-Net, and DeepLabV3 + in classification power.

### Ablation study

**Table 3:** Ablation study

Model Configuration	Accuracy	Precision	Recall	F1-Score	Dice Score	IoU
SegNet-only + MLP	0.8923	0.9015	0.8958	0.8986	0.78	0.65
SegNet + ResNet-50 + MLP	0.9216	0.9289	0.9312	0.93	0.83	0.72
SegNet + ResNet-50 + ViT	0.9351	0.9453	0.9407	0.943	0.84	0.75
SegResDeiT (Proposed)	0.9447	0.9566	0.9647	0.9606	0.86	0.79

An extensive ablation study was conducted to objectively evaluate the contribution of each component in our hybrid architecture, with the results shown in Table 3. The baseline model, using only SegNet with an MLP classifier, achieved a notable accuracy of 89.23% and a Dice score of 0.78. Incorporating ResNet-50 for deep feature extraction led to a significant improvement, increasing accuracy to 92.16% and the Dice score to 0.83, underscoring the importance of hierarchical feature learning. Replacing the MLP head with a conventional Vision Transformer (ViT) to include global context modelling further enhanced performance, reaching an accuracy of 93.51% and a Dice score of 0.84. Ultimately, our proposed SegResDeiT architecture, which employs the data-efficient DeiT transformer, delivered superior results across all metrics, with an accuracy of 94.47%, a precision of 0.9566, a recall of 0.9647, an F1-score of 0.9606, and a Dice coefficient of 0.86. This progressive development demonstrates that each element SegNet’s spatial precision, ResNet-50’s rich feature extraction, and DeiT’s advanced contextual understanding offers unique and complementary benefits, with DeiT’s distillation approach proving particularly effective for our medical imaging dataset compared to the traditional ViT.

### Computational efficiency analysis

**Table 4:** Computational efficiency analysis of different architectures

Model	Parameters (M)	GFLOPs	Inference Time (ms)	Dice Score
SegResDeiT (Proposed)	52.3	18.4	47 ± 3.2	0.9606
Swin-UNet	61.8	22.1	53 ± 4.1	0.8971
nnU-Net	49.1	16.9	42 ± 2.8	0.8471
DeepLabV3 + with(ResNet-50)	39.2	12.7	38 ± 2.5	0.8322

Model performance and efficiency were evaluated on an NVIDIA RTX 3080 GPU, using metrics for a common 256 × 256 × 3 input image. In Table 4, the SegResDeiT framework achieves the highest Dice Score (0.9606), indicating superior classification accuracy. With a moderate computational cost of 52.3 million parameters and 18.4 GFLOPs, this performance achieves an inference time of 47 ± 3.2 milliseconds. While Swin-UNet is a strong transformer-based alternative, it takes more computational resources (61.8 M parameters, 22.1 GFLOPs) and a longer inference time (53 ± 4.1 ms) with a lower Dice Score (0.8971). Among top performers, the nnU-Net framework achieves a balance between parameter count (49.1 M) and GFLOPs (16.9), with an inference time of 42 ± 2.8 ms and a Dice Score of 0.8471. While DeepLabV3 + with ResNet-50 is the most efficient model (39.2 M parameters, 12.7 GFLOPs, 38 ± 2.5 ms inference time), it also has the lowest Dice Score (0.8322) among related models. These findings suggest that SegResDeiT offers the optimal balance between state-of-the-art classification accuracy and realistic computational requirements for clinical integration.

### Discussion

The extensive experimental investigation indicates that the proposed SegResDeiT architecture represents a notable improvement in the automated segmentation and classification of cervical cancer from cytological pictures. The model’s exceptional performance, evidenced by its leading metrics of accuracy (94.47%), precision (95.66%), recall (96.47%), F1-score (96.06%), IoU (92.43%), and Dice coefficient (96.06%), unequivocally demonstrates its superiority over notable baseline models such as Swin-UNet, nnU-Net, and DeepLabV3+. The performance superiority is not only quantitative but is also supported by visual evidence, demonstrating a significant alignment between the predicted segmentation masks and the expert-annotated ground facts, precisely outlining lesion boundaries with minimum inaccuracies. The elevated recall is especially vital in medical screening, as it reflects the model’s efficacy in reducing false negatives, so guaranteeing that probable abnormalities are detected.

The confusion matrix and ROC curve evaluations offer enhanced understanding of the model’s dependability. SegResDeiT demonstrated the most equitable classification performance, with the lowest rates of false positives and false negatives across all compared models. The ROC curve, closely adhering to the top-left corner with an AUC of 0.9314, demonstrates exceptional discriminatory capability in differentiating tumor from non-tumor patients. The elevated sensitivity and specificity are crucial for a decision-support tool designed for clinical settings, where diagnostic precision has a direct impact on patient outcomes. Conversely, the alternative models, albeit proficient, exhibited increasingly elevated misclassification rates and diminished AUC values, underscoring a significant disparity in generalization and dependability that SegResDeiT effectively addresses.

The ablation investigation provides strong support for the synergistic design decisions inside the SegResDeiT framework. The incremental enhancement in performance from a SegNet-MLP baseline to the integration of ResNet-50’s robust feature extraction, and ultimately to the inclusion of the Data-efficient Image Transformer (DeiT) affirms the significance of each element. The significant performance enhancement realized by substituting a conventional MLP head with the DeiT transformer highlights the essential role of capturing global contextual relationships for this task. The higher performance of DeiT compared to a standard ViT underscores the efficacy of its distillation mechanism in acquiring robust representations from the restricted data characteristic of medical imaging datasets, rendering it both potent and pragmatically appropriate. Moreover, the investigation of computing efficiency establishes SegResDeiT as a plausible choice for inclusion into clinical practice. The model achieves optimal accuracy while preserving a manageable computational footprint. While it is not the most lightweight model, as nnU-Net and DeepLabV3 + with ResNet-50 possess fewer parameters and exhibit quicker inference times, it achieves an ideal equilibrium. A significant improvement in diagnostic efficacy warrants the somewhat increased computational expense relative to these models.

In comparison to the Swin-UNet transformer-based approach, SegResDeiT demonstrates superior accuracy and efficiency, necessitating fewer parameters and GFLOPs. This equilibrium is crucial for practical implementation in healthcare environments, where resources may be limited yet diagnostic accuracy must remain intact. In conclusion, the discourse confirms that the SegResDeiT model constitutes a formidable and effective alternative for cervical cancer screening. Its hybrid architecture effectively utilizes the advantages of convolutional networks for spatial feature extraction and contemporary transformers for global context comprehension. The comprehensive empirical validation substantiates its superiority in segmentation precision, classification dependability, and operational feasibility. Future endeavours will concentrate on external validation utilising multi-centre datasets and investigating the model’s adaptability to additional cancer diagnostics, ultimately aiming to enhance early and precise detection to increase patient care. Our results indicate robust performance on the APACS23 dataset; nonetheless, we recognize certain limitations. The research employed a single-centre dataset, necessitating external validation on multi-institutional data to evaluate generalizability across diverse staining techniques, scanner discrepancies, and demographic variances. Future endeavours will encompass validation on bigger, multi-centre cohorts and the investigation of domain adaptation methodologies to improve robustness. Furthermore, although we conducted thorough internal validation using a hold-out test set, prospective clinical validation is necessary to confirm real-world efficacy.

## Conclusion

In conclusion, the SegResDeiT model introduced in this study has effectively showcased its capability as a precise and dependable instrument for the automated segmentation and classification of cervical cancer. The thorough assessment clearly demonstrates the superiority of this hybrid architecture, which strategically combines the spatial accuracy of a SegNet encoder-decoder framework with the intricate feature hierarchies of ResNet-50 and the global contextual comprehension of a Data-efficient Image Transformer (DeiT). The model has remarkable performance, attaining a Dice coefficient of 96.06%, an IoU of 92.43%, and a classification accuracy of 94.47%, thereby surpassing existing benchmarks such as Swin-UNet, nnU-Net, and DeepLabV3+. The high recall of 96.47% underscores the model’s key capability to minimise false negatives, a fundamental requirement for any screening method aimed at early disease diagnosis. The architectural design’s validity is reinforced by the ablation study, which verified the synergistic role of each component, with the DeiT module being especially crucial for capturing long-range relationships that improve diagnostic accuracy. In addition to its raw accuracy, the model’s practical utility is enhanced by its computing efficiency. SegResDeiT achieves cutting-edge findings while maintaining a manageable computing footprint, effectively balancing performance and resource requirements crucial for clinical applicability. The visual outcomes, demonstrating significant concordance with expert annotations, along with the comprehensive ROC analysis (AUC = 0.9314), furnish compelling evidence of the model’s generalization ability and dependability. This paper proposes a novel technical framework and presents a viable approach to addressing significant issues in global cervical cancer screening. The SegResDeiT model enhances the speed, consistency, and precision of cytology image processing, potentially easing the strain on healthcare systems, diminishing inter-observer variability, and promoting earlier intervention. Future initiatives will focus on external validation using varied, multi-institutional datasets and examining the model’s applicability to other oncological fields, ultimately facilitating its incorporation into computer-aided diagnostic systems to enhance global patient outcomes.

## Data Availability

All data generated or analysed during this study are included in this published article “Cell Segmentation in Digitized Pap Smear Images Using an Ensemble of Fully Convolutional Networks”.
